# NADPH Oxidase Overactivity Underlies Telomere Shortening in Human Atherosclerosis

**DOI:** 10.3390/ijms21041434

**Published:** 2020-02-20

**Authors:** Álvaro Pejenaute, Adriana Cortés, Javier Marqués, Laura Montero, Óscar Beloqui, Ana Fortuño, Amelia Martí, Josune Orbe, Guillermo Zalba

**Affiliations:** 1 Department of Biochemistry and Genetics, University of Navarra, 31008 Pamplona, Spain; apejenaute@alumni.unav.es (Á.P.); acortes.3@alumni.unav.es (A.C.); jmarques.1@alumni.unav.es (J.M.); monterobarreras@gmail.com (L.M.); 2 Navarra Institute for Health Research (IdiSNA), 31008 Pamplona, Spain; obeloqui@unav.es (Ó.B.); amarti@unav.es (A.M.); josuneor@unav.es (J.O.); 3 Department of Internal Medicine, Clínica Universidad de Navarra, 31008 Pamplona, Spain; 4 Program of Cardiovascular Diseases, CIMA, University of Navarra, 31008 Pamplona, Spain; afortuno@unav.es; 5 Department of Food Sciences and Physiology, University of Navarra, 31008 Pamplona, Spain

**Keywords:** atherosclerosis, intima-media thickness, NADPH oxidase, oxidative stress, telomere length

## Abstract

Telomere shortening and oxidative stress are involved in the pathogenesis of atherosclerosis. Different studies have shown that phagocytic NADPH oxidase is associated with this disease. This study aimed to investigate the association between phagocytic NADPH oxidase and telomere shortening in human atherosclerosis. To assess this potential association, telomere length and phagocytic NADPH oxidase activity were determined by PCR and chemiluminescence, respectively, in a population of asymptomatic subjects free of overt clinical atherosclerosis. We also measured serum 8-hydroxy-2-deoxyguanosine (8-OHdG) levels (an index of oxidative stress) and carotid intima-media thickness (IMT), a surrogate marker of atherosclerosis. After adjusting them for age and sex, telomere length inversely correlated (*p* < 0.05) with NADPH oxidase-mediated superoxide production, with 8-OHdG values, and with carotid IMT. Interestingly, the asymptomatic subjects with plaques have a lower telomere length (*p* < 0.05), and higher values of plasma 8-OHdG and superoxide production (*p* < 0.05). These data were confirmed in a second population in which patients with coronary artery disease showed lower telomere length and higher 8-OHdG and superoxide production than the asymptomatic subjects. In both studies, NADPH oxidase-dependent superoxide production in phagocytic cells was only due to the specific expression of the Nox2 isoform. In conclusion, these findings suggest that phagocytic NADPH oxidase may be involved in oxidative stress-mediated telomere shortening, and that this axis may be critically involved in human atherosclerosis.

## 1. Introduction

Atherothrombosis is associated with aging and constitutes the main cause of death in Western and developing countries [1]. It comprises of, firstly, the formation and growth of atherosclerotic plaques, and secondly, the rupture of plaques, which constitutes the key trigger of cardiovascular diseases including myocardial infarction and stroke. The pathological mechanisms involved in this particular process include, among others, endothelial dysfunction, vascular proliferation, inflammation, matrix degradation and apoptosis, and thrombosis [2]. Oxidative stress, defined as an imbalance between oxidants and antioxidants in favour of the former, participates both in the early stages and in the late phases of the atherothrombotic process [3,4,5].

The NADPH oxidase family represents the most important vascular source of reactive oxygen species (ROS), mainly superoxide and hydrogen peroxide, which are present in endothelial cells, smooth muscle cells, fibroblasts, and infiltrated monocytes/macrophages [6,7]. Vascular NADPH oxidase isoforms (Nox1, 2, 4 and 5) are involved in human atherosclerosis [8,9,10,11]. The phagocytic NADPH oxidase isoform (Nox2) also participates actively in the development and progression of the atherosclerotic lesion [11,12,13], mainly from monocytes/macrophages. Interestingly, the activity of the blood phagocytic NADPH oxidase correlates positively with carotid intima-media thickness (IMT) [14] (a surrogate marker of atherosclerosis), and with matrix metalloproteinase-9 plasma levels [15] (a marker of vascular remodelling and an independent risk factor for atherothrombotic events), in healthy subjects without clinical atherosclerosis.

Telomeres are chromatin regions dedicated to the maintenance of chromosome integrity. They are located at the end of the chromosome arms and contain a high number of non-coding repetitions (5′-TTAGGG-3′ in vertebrates), reaching up to 15,000 base pairs (bp) in humans [16]. Telomere length has been suggested as a biomarker of chronic oxidative stress [17]. Telomere shortening in vascular cells, including infiltrated phagocytic cells in atherosclerotic plaques, requires an imbalance in ROS homeostasis [18]. Short telomeres are associated with increased carotid atherosclerosis in hypertensive subjects [19]. Telomere length is diminished in the white blood cells of atherosclerotic patients [20]. Moreover, type-2 diabetes patients presenting atherosclerotic plaques exhibit shorter telomeres than those without plaques [21]. Furthermore, oxidative stress-induced telomere shortening might represent a key predictor of cardiovascular events. In this study, we hypothesize that the increased NADPH oxidase-mediated superoxide production in phagocytic cells may be favouring telomere shortening, which might be a feature of atherosclerosis. In order to test this hypothesis, we explored the relationship of NADPH oxidase-mediated superoxide production in phagocytic cells with telomere length in subclinical (a general population of asymptomatic individuals with assessed carotid IMT) and clinical (patients with coronary artery disease -CAD-) atherosclerosis.

## 2. Results

### 2.1. Cohort 1

*Clinical characteristics of the individuals.* The demographic and clinical characteristics of the studied subjects are summarized in Table 1.

The group presenting plaques in their carotid arteries were significantly older than the subjects with no plaques. The group with carotid plaques displayed significantly higher systolic blood pressure (SBP) and plasma levels of glucose than the control group. In addition, the group of individuals with plaques presented an increased carotid IMT compared with the control group. No significant differences were found in the remaining parameters between the two groups of subjects. Finally, we found remarkable differences in the frequency of cardiovascular medications (antihypertensives, statins and hypoglycemic) between the two groups.

**Figure 1:**
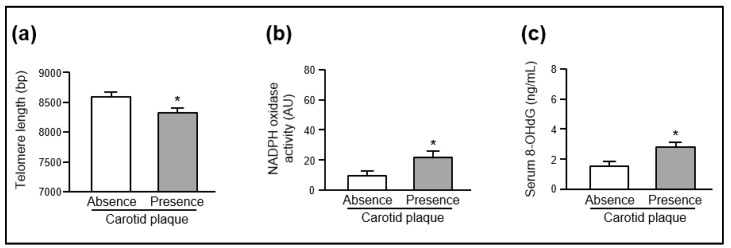
(**a**) Telomere length in circulating leucocytes, (**b**) NADPH oxidase-dependent superoxide production in peripheral blood mononuclear cells, and (**c**) serum 8-OHdG levels in asymptomatic individuals, according to the absence (*n* = 389) or presence (*n* = 116) of atherosclerotic plaques in carotid arteries. * *p* < 0.05 after adjusting for age and sex. bp, base pairs; AU, arbitrary units; 8OHdG, 8-hydroxy-2-deoxyguanosine.

*Telomere length.* As shown in Figure 1a, the length of circulating telomere leucocytes was lower (*p* < 0.05) in individuals presenting plaques than in control subjects (Presence: 8315 ± 98 bp; Absence: 8591 ± 84 bp). These differences remained statistically significant when telomere length was adjusted for age and sex.

*NADPH oxidase activity and serum levels of 8-hydroxy-2-deoxyguanosine*. As shown in Figure 1b, NADPH oxidase-mediated superoxide production in peripheral mononuclear cells was higher (*p* < 0.05) in the group of individuals presenting plaques than in the control group (Presence: 21.1 ± 2.9 AU; Absence: 9.5 ± 2.5 AU). Serum levels of 8-hydroxy-2-deoxyguanosine (8-OHdG) were higher (*p* < 0.05) in the individuals presenting plaques than in the control group (Presence: 2.8 ± 0.3 ng/mL; Absence: 1.5 ± 0.3 ng/mL) (Figure 1c). These differences remained statistically significant after adjusting for age and sex.

**Figure 2:**
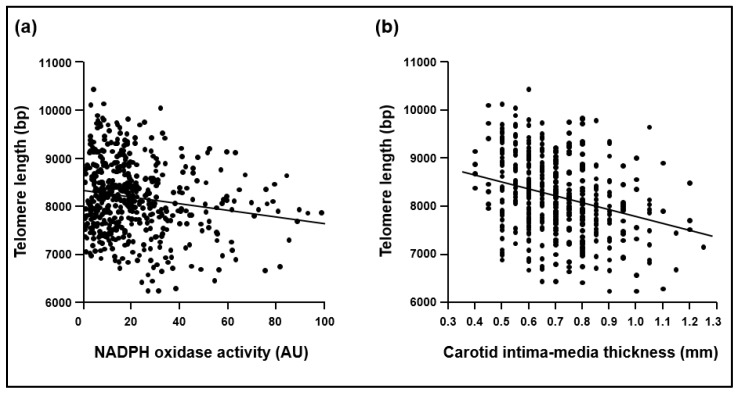
Inverse correlation of telomere length with (**a**) NADPH oxidase activity (R = −0.255, *p* < 0.001 after correcting it for age and sex) and (**b**) carotid intima-media thickness (R = −0.173, *p* < 0.001 after correcting it for age and sex) in all the asymptomatic population. bp, base pairs. AU, arbitrary units.

*Analysis of associations.* There was a noticeable negative bivariate correlation between the telomere length and age in all subjects (R = −0.236, *P*<0.001). The telomere length negatively correlated with NADPH oxidase-dependent superoxide generation (R = −0.173, *p* < 0.001) after correcting it for age and sex (Figure 2), and with 8-OHdG levels (R = −0.187, *p* < 0.001). Finally, as expected, the telomere length also negatively correlated with the carotid IMT (Figure 2).

### 2.2. Cohort 2

*Clinical characteristics of the individuals.* The demographic and clinical characteristics of the studied subjects are summarized in Table 2. The CAD patients displayed appreciably higher SBP and plasma levels of glucose than the control subjects. In addition, the group of individuals with plaques showed an increased carotid IMT compared to the control group. There were no significant differences in the remaining parameters between the two groups of subjects.

**Figure 3:**
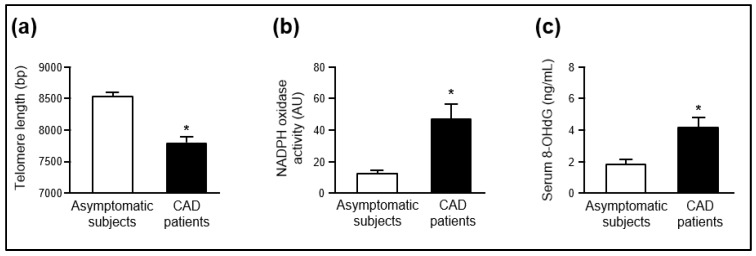
(**a**) Telomere length in leucocytes, (**b**) NADPH oxidase-dependent superoxide production in peripheral blood mononuclear cells, and (**c**) serum 8-OHdG levels in asymptomatic individuals (*n* = 25) and CAD patients (*n* = 25). * *p* < 0.05 compared with asymptomatic subjects after adjusting for age and sex. CAD, coronary artery disease. bp, base pairs; AU, arbitrary units; 8-OHdG, 8-hydroxy-2-deoxyguanosine.

*Telomere length.* As shown in Figure 3a, the telomere length in circulating leucocytes was lower (*p* < 0.05) in the group of CAD patients than in the control group (CAD group: 7784 ± 113 bp; control group: 8527 ± 71 bp). These differences remained statistically significant when the telomere length was adjusted for age and sex.

*Phagocytic NADPH oxidase activity and serum 8-OHdG*. As shown in Figure 3b, NADPH oxidase-mediated superoxide production in peripheral mononuclear cells was higher (*p* < 0.05) in the CAD group than in the control group (CAD group: 47.1 ± 8.9 AU; control group: 12.1 ± 2.1 AU). Interestingly, serum levels of 8-OHdG were higher (*p* < 0.05) in the CAD group than in the control group (CAD group: 4.2 ± 0.6 ng/mL; control group: 1.8 ± 0.3 ng/mL) (Figure 3c). These differences remained statistically significant after adjusting for age and sex.

### 2.3. Characterization of NADPH Oxidase Isoforms Involved in NADPH Oxidase Activity in Asymptomatic Individuals and CAD Patients

**Figure 4:**
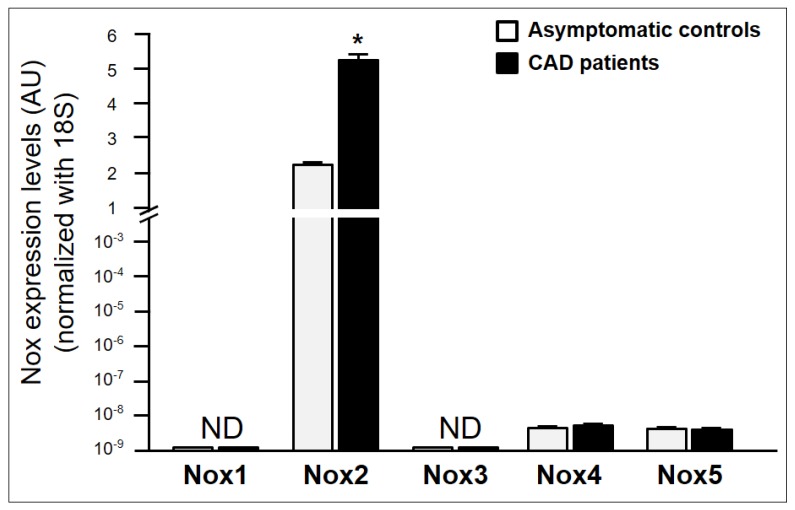
NADPH oxidase subunits Nox1, Nox2, Nox3, Nox4 and Nox5 mRNA levels in peripheral blood mononuclear cells from asymptomatic individuals (*n* = 147) and in CAD patients (*n* = 25). * *p* < 0.05 compared with control subjects. CAD, coronary artery disease; AU, arbitrary units; ND, non-detected. Nox, NADPH oxidase.

In our study, we analysed the expression of Nox1-5 in the peripheral mononuclear cells from both studied cohorts. As presented in Figure 4, we detected relevant levels of expression for Nox2. As expected, the levels of Nox2 were significantly higher (*p* < 0.05) in the group of CAD patients than in the control group (CAD group: 5.25 ± 0.22 AU; control group: 2.21 ± 0.11 AU). The expression levels of Nox1, Nox3, Nox4 and Nox5 were absent or practically negligible both in the asymptomatic control group and in the CAD group.

Within the group of asymptomatic subjects, individuals presenting atherosclerotic plaques exhibited a greater expression of Nox2 (absence of plaques: 1.6 ± 0.1 AU; presence of plaques: 3.8 ± 0.2 AU, *p* < 0.05). Nevertheless, we did not detect either Nox1 or Nox3, and the levels of Nox4 and Nox5 were practically trivial.

## 3. Discussion

The main findings of this study are: (i) in a population of asymptomatic individuals, free of clinically evident atherosclerotic disease, telomere attrition associates with increased phagocytic NADPH oxidase-dependent superoxide production, serum 8-OHdG levels (markers of oxidative stress), and enhanced carotid IMT (a surrogate index of subclinical atherosclerosis); (ii) in this asymptomatic population, individuals with carotid plaques presented higher levels of telomere shortening, superoxide production, and 8-OHdG; (iii) patients with CAD presented maximum telomere shortening, superoxide production generation, and 8-OHdG levels; and (iv) in both populations, NADPH oxidase-dependent superoxide production was only due to the specific expression of the Nox2 isoform. These findings support a relevant role of Nox2 in oxidative stress-mediated telomere shortening, and highlights an important relationship that may be critically involved in human atherosclerosis.

Numerous studies support that telomere shortening is closely associated with the pathogenesis and the severity of atherosclerosis. Firstly, human atherosclerotic plaques present shorter telomeres than normal vessels. Smooth muscle cells from fibrous cap exhibit shorter telomeres compared to normal medial cells [18]. Secondly, type-2 diabetes patients presenting atherosclerotic plaques display shorter telomeres than those without plaques [21]. Thirdly, the more pronounced leucocyte telomere attrition found in adult atherosclerotic patients seems to be mainly due to increased attrition in early life [22]. Finally, most epidemiological studies support a relevant role of leucocyte telomere shortening as a cause for atherosclerotic vascular diseases [23,24]. Accordingly, our findings show greater telomeric shortening in asymptomatic subjects who have atherosclerotic plaques versus those who do not (cohort 1), which consequently supports that the telomere attrition should play a fundamental role in atherogenesis. Moreover, symptomatic patients with CAD (cohort 2) present maximum telomere attrition.

Numerous findings support that oxidative stress is related to telomere shortening [25]. Oxidative stress triggers telomere attrition in endothelial cells [26] and vascular smooth muscle cells [27,28]. Furthermore, monocytes from type 2 diabetes patients exhibit telomere shortening and increased oxidative DNA damage [20]. In addition to this, chronic treatment with N-acetyl-cysteine, a scavenger of ROS, delays cellular senescence in endothelial cells isolated from atherosclerotic patients [29]. In this respect, several studies support that ROS boost vascular telomere length reduction in atherosclerotic plaques [18,30,31]. Suitably, our results show that paired to the initiation and progression of atherosclerotic disease, there is a progressive shortening of leucocyte telomeres accompanied by an increase in circulating mononuclear cell NADPH oxidase activity and in oxidative DNA damage. In fact, the telomere length inversely correlates with 8-OHdG and with NADPH oxidase activity. Therefore, it could be useful to speculate that pro-atherosclerotic humoral factors may have, in fact, an effect on the phagocytic NADPH oxidase in patients with atherosclerosis.

Nox-mediated ROS could be involved in both telomere shortening and cellular senescence [32]. On one hand, telomere length is reduced in Nox4-depleted HUVECs [33]. On the other hand, the life span of Nox4-deficient mice is similar to their wild type littermates [34]. In this latter study, neither telomerase (TERT) expression nor telomere length presented differences in endothelial cells isolated from the lungs of those animals. Previous studies have shown that NADPH oxidases are crucially involved in atherosclerosis [8,9,10,11,12]. On that account, infiltrated monocytes and macrophages are responsible for high levels of Nox-mediated ROS in human atherosclerotic plaques [9,12]. Thus, we cannot discard that NADPH oxidase-dependent oxidative stress from infiltrated phagocytic cells might be involved in the promotion of telomere imbalance in themselves and/or in neighbouring cells. In agreement with this, senescent intimal foam cells are deleterious at all stages of atherosclerosis [35]. As we have previously demonstrated, phagocytic NADPH oxidase activity correlates with surrogate markers of atherosclerosis, including carotid IMT [14], plasma levels of matrix metalloproteinase-9 [15], and coronary artery calcium [36]. In the present study, we point to a potential novel mechanism that suggests that the activation of this oxidase may participate in the progression towards worse atherosclerotic complications. Our findings, which show the association between NADPH oxidase overactivity and telomere shortening, support the potential involvement of this oxidase in vascular senescence. Moreover, in a study performed with 727 patients who were undergoing coronary artery bypass grafting, the additive effect of two functional polymorphisms in the *CYBA* locus, encoding the NADPH oxidase p22phox subunit, proved to be associated with increased phagocytic superoxide production and with blood telomere shortening [29,37].

Many studies have demonstrated that the upregulation of NADPH oxidases correlates with the increased production of ROS [38,39]. Nox2 is the traditional isoform expressed constitutively in white blood cells. Recently, several papers have insisted upon a notorious role for other NOXes, including Nox4 [40] and Nox5 [41] isoforms, which appear to be upregulated in circulating mononuclear cells (monocytes) in an atherogenic environment. In this context, our findings show that the phagocytic NADPH oxidase activity is due to the expression of the Nox2 isoform, both in the controlled asymptomatic subjects and in atherosclerotic patients. This supports the major importance of the Nox2 isoform ROS generation in circulating white blood cells in atherogenesis, without ignoring that the upregulation of other Nox isoforms in phagocytes could be significantly involved in the subsequent steps of the atherothrombotic process.

In conclusion, our research suggests that the phagocytic NADPH oxidase is associated with telomere shortening both in asymptomatic and symptomatic human atherosclerosis. This study adds new data on the role of oxidative stress in the pathophysiology of atherosclerosis and allows us to suggest a relevant implication of the phagocytic NADPH oxidase in telomere shortening, a non-traditional risk factor, which has been proposed as an independent and additive predictor of adverse cardiovascular outcomes in CAD patients [42].

## 4. Materials and Methods

### 4.1. Human Studies

The study was designed according to the ethical standards of our institution, and following the Declaration of Helsinki. The Ethical Committee of the University Clinic of Navarra approved the study protocol on 27 May 2010 (No. 086/2010, “Telomeric shortening and atherosclerosis in metabolic syndrome patients: therapeutic effect of the blockade of systemic oxidative stress”) and informed consent was obtained from each subject involved in the study.

Cohort 1. This population was comprised of 505 apparently healthy middle-aged individuals, who were recruited in our institution after a routine 12-h fasting medical check-up. The clinical screenings included medical history, physical examination, and analytical tests. Carotid IMT and atheroma plaques were determined by ultrasonography, as previously described [43]. Carotid IMT was measured over 1-cm length, proximal to the carotid bulb of each common carotid artery. Plaques were defined as local enlargements of the carotid IMT with an area 50% greater than the IMT of neighbouring sites.

Cohort 2. Twenty-five consecutive patients undergoing coronary angiography in our institution with clear symptoms of CAD were eligible for inclusion in the study (21 men, aged 62 years, 68% hypertensive, 25% hyperlipidemic, 44% diabetic, 72% past and current smokers). Twenty-five controls were recruited from a screening program that was performed at the same time among the population in the area under our care. The control subjects were randomly selected from the screened individuals with no clinical symptoms of cardiovascular disease, including CAD.

### 4.2. Length of Telomeres

Leucocyte genomic DNA was extracted from human peripheral blood samples by using a QIAamp DNA Blood kit (Qiagen, Hilden, Germany). The telomere length was measured by real-time quantitative PCR [44,45], using the QuantiTect Syber Green PCR kit (Qiagen). This approach employs the Ribosomal Protein Large PO (RPLPO) single-copy gene as a reference for each sample. The measurements were performed on the ABI 7000 Sequence Detection System (Applied Biosystems, Thermo Scientific, Rockford, IL, USA). The total reaction volume was 20 μL and contained 30 ng of genomic DNA. The PCR mixes for the amplification of telomeres (T) and the single-copy gene (S) were identical except for the oligonucleotide primers. The final telomere primer concentrations were: tel1 270 nmol/L and tel2 900 nmol/L, for T amplification; and hRPLPO1 400 nmol/L and hRPLPO2 400 nmol/L, for S amplification. The primer sequences were tel1 (5′-GGTTTTTGAGGGTGAGGGTGAGGGTGAGGGTGAGGGT-3′), tel2 (5′-TCCCGACTATCCCTATCCCTATCCCTATCCCTATCCCTA-3′), hRPLPO1 (5′-CCCATTCTATCATCAACGGGTACAA-3′) and hRPLPO2 (5′-CAGCAAGTGGGAAGGTGTAATCC -3′). This method normalizes T to S by calculating the ratio (T/S ratio) for each sample. The T/S ratio was calculated as follows [2^CT(telomeres)^/2^CT(single copy gene)^] = 2^−ΔCT^.

We prepared a calibration curve with an intact genomic DNA sample, in 2-fold dilutions from 64 to 0.25 ng, which was used as a standard in all amplifications in order to control the day-to-day variations. A linearity R^2^ > 0.98 for the standard curve was considered acceptable. We ran all samples in triplicate. The samples presenting a variation among the triplicates >10% were rerun and reanalysed. The intra-assay coefficient of variation between the triplicates was 2.2% and the inter-assay coefficient of variation between plates was 1.8%, which supports the power of this procedure. PCR results, in arbitrary units, were converted into base pairs by using an extrapolating line generated with a reduced pool of 20 different DNA samples.

### 4.3. Determination of Superoxide Production in Human Peripheral Blood Cells

We isolated mononuclear cells from the blood samples with Lymphoprep. Four hundred thousand cells were used to measure NADPH oxidase-dependent superoxide production in response to stimulation with 3.2 μmol/L phorbol myristate acetate (Sigma Aldrich), and by using 5 μmol/L lucigenin (Sigma Aldrich) in a plate reading luminometer (Luminoskan Ascent, Labsystem, Thermo Scientific) [46,47]. Measurements of 1 s were recorded every 15 s with an interval of 60 min in the luminometer. The integrated value of the area under the curve was used to quantify chemiluminescence, which was expressed as arbitrary units (AU). These chemiluminescence measurements correlated with an independent measurement of superoxide production using superoxide dismutase (SOD)-inhibitable ferricytochrome c reduction. Our group has previously featured this protocol with the use of Cu,Zn-SOD (an enzymatic scavenger of superoxide), and different inhibitors, including diphenylene iodonium (an inhibitor of flavoproteins), apocynin and gp91ds-tat (inhibitors of NADPH oxidase assembly), rotenone (an inhibitor of the mitochondrial chain), oxypurinol (an inhibitor of the xanthine oxidase), and bisindolyl maleimide (an inhibitor of protein kinase C) [47].

### 4.4. RNA Extraction and Real-Time Quantitative Polymerase Chain Reaction

After obtaining mononuclear cells from the blood samples with Lympoprep, the total RNA was isolated from these cells by using TRIzol reagent (Invitrogen, Thermo Scientific). One microgram of RNA was used to perform the reverse transcription using the RNA SuperScript VILO^TM^ cDNA Synthesis Kit (Invitrogen, Thermo Scientific). Quantification of the cDNA was performed by real-time PCR with specific TaqMan MGB fluorescent probes (Hs00246589_m1 for Nox1, Hs00166163_m1 for Nox2, Hs00210462_m1 for Nox3, Hs00418356_m1 for Nox4, Hs00225846_m1 for Nox5, and 4333760F for 18S ribosomal RNA, Applied Biosystems, Thermo Scientific) in an ABI Prism 7000 Sequence Detection System (Applied Biosystems, Thermo Scientific). Data were expressed as AU relative to the 18S ribosomal RNA. PCR cycles were as follows: initial denaturation for 30 s at 95°C, followed by 40 cycles at 95 °C for 5 s and 60 °C for 30 s. The relative index of gene expression was calculated by using the 2^−ΔΔCt^ method.

### 4.5. Measurement of 8-hydroxy-2-deoxyguanosine Levels

Serum 8-OHdG levels were measured with the OxiSelect^™^ Oxidative DNA Damage ELISA Kit (Cells Biolabs, Madrid, Spain) according to the manufacturer’s instructions.

### 4.6. Statistical Analysis

Statistical analyses were performed using SPSS 20 (SPSS, Inc., Chicago, IL, USA). The continuous variables were expressed as mean±SEM, and bivariate data as frequencies and percentages. Comparisons of continuous variables between groups were assessed by one-way ANOVA once normality was demonstrated, followed by a Scheffé post hoc test. If not, a Kruskal–Wallis followed by a Mann–Whitney U test was used instead. Comparisons between groups for categorical variables were performed with a χ^2^ test. The correlation between continuous variables was evaluated with Pearson’s correlation test. *p* < 0.05 was considered statistically significant.

## Figures and Tables

**Table 1:** Demographical and clinical characteristics of the **cohort 1** (asymptomatic subjects) according to the presence/absence of atherosclerotic plaques in carotid arteries.

Parameters	Atherosclerotic Plaques in Carotid Arteries	
	Absence (*n* = 389)	Presence (*n* = 116)
Age, year	54 ± 1	61 ± 1 *
Male Gender, %	80	88*
BMI, kg/m^2^	28.8 ± 0.2	28.9 ± 0.5
SBP, mmHg	127 ± 1	137 ± 2 *
DBP, mmHg	82 ± 1	83 ± 2
Glucose, mg/dL	98 ± 1	107 ± 2 *
HDL-cholesterol, mg/dL	49 ± 1	51 ± 2
LDL-cholesterol, mg/dL	145 ± 3	139 ± 5
Total cholesterol, mg/dL	219 ± 4	212 ± 3
Triglycerides, mg/dL	117 ± 3	113 ± 5
Carotid IMT, mm	0.67 ± 0.01	0.79 ± 0.02 *
Medication		
Antihypertensives, %	15	37 *
Statins, %	16	31 *
Oral hypoglycemics, %	8	16 *

BMI, body mass index; HDL, high-density lipoprotein; LDL, low-density lipoprotein; SBP, systolic blood pressure; DBP, diastolic blood pressure; IMT, intima-media thickness. * *p* < 0.05.

**Table 2:** Demographical and clinical characteristics of the **cohort 2**.

Parameters	Control Subjects (*n* = 25)	CAD Patients (*n* = 25)
Age, year	59 ± 2	62 ± 3
Male Gender, %	80	80
BMI, kg/m^2^	28.7 ± 1.6	28.3 ± 1.7
SBP, mmHg	128 ± 2	138 ± 2 *
Glucose, mg/dL	99 ± 2	111 ± 2 *
HDL-cholesterol, mg/dL	51 ± 3	53 ± 3
LDL-cholesterol, mg/dL	124 ± 14	98 ± 12
Total cholesterol, mg/dL	205 ± 18	178 ± 16
Triglycerides, mg/dL	115 ± 12	110 ± 13
Arterial Hypertension, %	24	68*
Hyperlipidemia, %	20	24
Diabetes, %	12	44*

BMI, body mass index; HDL, high-density lipoprotein; LDL, low-density lipoprotein; SBP, systolic blood pressure. * *p* < 0.05.
